# When Bladder and Brain Collide: Is There a Gender Difference in the Relationship between Urinary Incontinence, Chronic Depression, and Anxiety?

**DOI:** 10.3390/jcm12175535

**Published:** 2023-08-25

**Authors:** Muhammed Furkan Dasdelen, Furkan Almas, Suleyman Celik, Nursanem Celik, Zuleyha Seyhan, Pilar Laguna, Selami Albayrak, Rahim Horuz, Mehmet Kocak, Jean de la Rosette

**Affiliations:** 1 International School of Medicine, Istanbul Medipol University, 34810 Istanbul, Türkiye; furkan.almas@std.medipol.edu.tr (F.A.); suleyman.celik@std.medipol.edu.tr (S.C.); zuleyha.seyhan@std.medipol.edu.tr (Z.S.); plaguna@medipol.edu.tr (P.L.); rhoruz@medipol.edu.tr (R.H.); mehmetkocak@medipol.edu.tr (M.K.); 2 School of Medicine, Istanbul Medipol University, 34810 Istanbul, Türkiye; nursanem.celik@std.medipol.edu.tr (N.C.); salbayrak@medipol.edu.tr (S.A.); 3 Department of Urology, Istanbul Medipol University, 34810 Istanbul, Türkiye; 4 Department of Biostatistics and Medical Informatics, Istanbul Medipol University, 34810 Istanbul, Türkiye

**Keywords:** urinary incontinence, depression, anxiety, cross-sectional study, Turkish, survey

## Abstract

In longitudinal and cross-sectional studies, depression and anxiety have been associated with urinary incontinence (UI) in women. However, this association has not been studied in men. Utilizing data from the 2008 Turkish Health Studies Survey conducted by the Turkish Statistical Institute, we analyzed 13,830 participants aged 15 years and above. We investigated the association of UI with psychological discomfort in both sexes using multivariable logistic regression. High psychological discomfort significantly correlated with UI in males (OR 2.30, 95% CI 1.43–3.71) and females (OR 2.78, 95% CI 1.80–4.29). Anxiety increased UI likelihood in females (OR 2.36, 95% CI 1.61–3.46) and males (OR 2.37, 95% CI 1.10–5.13). Depression related significantly to UI in females (OR 2.54, 95% CI 1.81–3.58) but not males (OR 1.63, 95% CI 0.71–3.76). Antidepressant and anxiolytic use was not significantly related to UI in either gender. Anxiety and psychological discomfort contribute to UI in both genders. While depression significantly correlates with UI in females, it does not show the same magnitude and significance in males. Antidepressant and anxiolytic use did not significantly influence the association. These findings underscore the psychological distress-UI link, advocating a holistic approach for managing UI in individuals with mental health conditions.

## 1. Introduction

Urinary incontinence (UI), characterized by involuntary leakage of urine, is a common distressing health problem. It can impair the quality of life and give rise to a range of psychosocial issues, such as anxiety, depression, and social isolation [1]. Urinary incontinence affects both sexes, but it is twice as prevalent in women [2]. The prevalence of UI in adult women varies greatly. The majority of studies reported a prevalence of any UI within the range of 25–45% [3,4]. In males, the occurrence rate of UI is approximately 3–11%, which escalates to a range of 11–31% in the older population [5,6,7,8]. Despite the limited number of studies in Turkiye, hospital-based surveys estimated that one in four Turkish women suffer from involuntary loss of urine [9].

The high prevalence of urinary incontinence not only affects the health-related quality of life but also increases individual and social health expenditures [10,11,12]. As UI is closely associated with age in both genders, the public burden of urinary incontinence is expected to rise in accordance with the current demographic trends [10,11,12]. Obviously, more attention should be given to factors that may increase the risk of urinary incontinence and may affect its prognosis.

Depression and anxiety are associated with UI in numerous longitudinal and cross-sectional studies [13,14,15,16,17,18,19,20,21]. This association is believed to be bidirectional, in which both conditions may increase each other’s prevalence. Urinary incontinence can cause embarrassment and limit daily activities, which in turn can result in feelings of depression and anxiety [22,23]. Conversely, disruption of the descending serotonergic system to the bladder caused by psychological distress may precipitate uncontrolled urine leakage and can affect the course and outcome of UI [21,22,24]. Neuropharmacological evidence indicates that certain depressive symptoms are correlated with diminished serotonergic functionality [25,26]. Serotonin-carrying nerve fibers descending from the brain form connections with sensory nerve endings, interneurons, and ganglia within the thoracolumbar and sacral spinal cords, all of which play a role in the act of voiding. Serotonergic input from the brain to sensory nerve endings in the dorsal section of the spinal cord inhibits sensory input. This could explain the direct suppression of bladder activity during filling and discomfort. Consequently, a reduction in serotonin levels that leads to a net reduction in inhibition might increase the susceptibility to unstable (overactive) contractions of the bladder, resulting in urinary incontinence [27,28]. Collectively, these observations suggest that a decline in serotonin function may predispose individuals to impaired mental health and contribute to urinary incontinence.

Several studies showing the correlation between UI, depression, and anxiety in the female population have been reported in the United States and northern European countries [12,13,20,22,23,29]. However, there is still a reporting gap for the prevalence of urinary incontinence in relation to the severity of psychological discomfort in men and in different geographical areas.

In this cross-sectional study, the primary aim was to explore the possible gender differences in the association of depression and anxiety with UI. Second, we aimed to study the correlation between psychological discomfort and urinary incontinence prevalence in both sexes. The third objective was to study the impact of antidepressant and anxiolytic use on the relationship between depression, anxiety, and urinary incontinence in men and women. Finally, we position the outcome of this Turkish survey toward similar international studies.

## 2. Material and Methods

This cross-sectional study is based on data from the 2008 Health Survey conducted by the Turkish Statistical Institute (TurkStat) on 1–18 April 2008. This population-based survey was undertaken as part of Eurostat’s European Health Interview Survey (EHIS) Wave 1 and included 20,624 participants from all age groups [30]. The survey aimed to monitor the health status, health care, and health determinants of the Turkish population, considering demographic and socioeconomic characteristics.

The survey modifications and interviews were conducted in accordance with EHIS regulations [31]. Before the main survey, a pre-test of the draft questionnaire was conducted in Ankara with the support of the Ankara Regional Office. This initial draft was revised with valuable insights from a committee formed of members from the Department of Public Health from Ankara University, Baskent University, Gazi University, Hacettepe University, and Gulhane Military Medical Academy, as well as related units of the Ministry of Health and other associated institutions. New modules were subsequently developed, tailored for children in the 0–6 and 7–14 age groups. A pilot application was then implemented through 26 TurkStat Regional Offices. The purpose of this pilot was to assess the field organization and to test the revised questionnaire form, especially after adding the new modules. Based on feedback and findings from the pilot, further revisions were made to the questionnaire.

The survey employs a two-stage stratified cluster sampling method to collect data from 7910 households in Turkiye, providing results that can be extrapolated for the entire country and allow for separate urban and rural estimations. The National Address Database, updated as of March 2008 and rooted in the 2007 Address Based Population Registry System, served as the sampling frame for this study. In this context, settlements with populations of 20,000 or less were defined as “rural”, whereas those with populations over 20,000 were termed “urban”. In the first stage of sampling, 372 clusters from urban areas and 233 from rural areas were selected, making a total of 605 clusters. For the subsequent stage, households were systematically chosen within each of these selected clusters.

Urban clusters had approximately 5580 households selected within the 372 identified clusters, with 15 households chosen systematically from each cluster. From these, interviews were successfully conducted with 4294 eligible households. Similarly, in the rural clusters, 2330 households were chosen from the 233 clusters, with 10 households from each cluster. Of these, 1846 eligible households were interviewed.

Weighting procedures were integral to this study to ensure that the data obtained could accurately represent the broader population. Initially, base weights, which are inversely proportional to overall selection probabilities, were computed for each respondent. This process entailed the calculation of selection probabilities for clusters and households. Following this, these base weights were adjusted to account for potential non-responses. Finally, the weights underwent calibration against the projected population distributions utilizing the integrated calibration ratio method, ensuring that any relative weight changes remained within a predefined scope [31].

The survey interviews were carried out face-to-face with the full consent of the participants. The microdata were made publicly available after all participants were anonymized.

### 2.1. Inclusion and Exclusion Criteria

First, we determined pre-specified risk factors and etiologies of UI, depression, and anxiety from previous studies to be included as adjustment variables in a logistic regression analysis.

Under the health status-related questions of the survey, the presence of various chronic diseases was asked for. Participants were expected to evaluate each condition separately and select one of four options as follows: (1) Yes, (2) No, (3) Don’t know, (4) Refusal. Participants who picked options 3 or 4 were considered to have a missing value and were excluded from further analysis. Outcome variables were utilized as patient-reported outcomes (PROs) regardless of a doctor’s confirmation or formal diagnosis history. We considered that the participant had the condition if they answered these questions as “Yes”.

**Figure 1:**
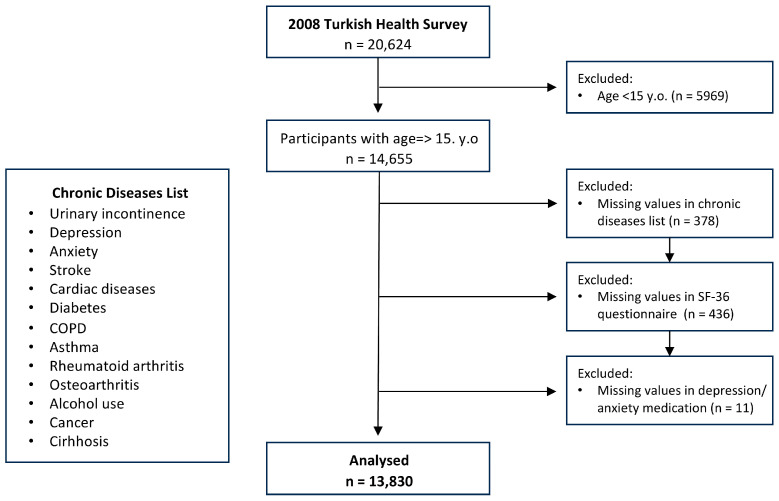
Inclusion and exclusion criteria of the study.

As shown in Figure 1, all participants were evaluated in terms of the independent variable list. Those who were under 15 years of age and who had missing values in any of the questions related to the presence of the chronic condition, psychological discomfort, and medication were excluded from the study. This resulted in 13,830 males and females.

### 2.2. Calculation of Psychological Discomfort Score and SF-36 Mental Health–Vitality Subscales

The survey included the psychological discomfort part of the 36-Item Short Form Health Survey questionnaire. The psychological discomfort score for each survey participant was calculated by adding up their responses. The psychological discomfort scale was divided into four categories based on data-driven quartile ranges: <20 is normal, between 20–23 is mild, 24–27 is moderate, and >27 is severe.

Transformed scores of mental health and vitality subscale calculations were made based on Ware et al. [32]. Briefly, items were recoded as defined in the manual. Raw scale scores were computed by summing across items in the same scale and were transformed to a 0–100 scale. All item recording and scale scoring were performed in Python 3.9.

### 2.3. Statistical Analysis

Descriptive statistics were used to characterize the study group regarding gender, urban-rural classification, UI, depression, anxiety, and other comorbidities as frequencies and percentages.

We used GraphPad for data visualization and related statistical analyses. The Statsmodels library in Python 3.9 was used for multivariate logistic regression models [33]. Three different logistic regression models with UI as an independent variable were used: (1) to investigate the association between UI and depression/anxiety, (2) to investigate the relationship between UI and psychological discomfort severity in the presence of other comorbidities, and (3) to understand the effect of psychotropic drugs on the UI-depression and UI-anxiety relationships. In all the logistic regression models, odds ratios were adjusted for age category, type of residency, alcohol consumption status, and comorbidities such as diabetes, cardiac diseases, stroke, rheumatoid arthritis, osteoarthritis, COPD, asthma, cancer, and cirrhosis.

The level of statistical significance was considered to be *p* < 0.05 throughout the study.

## 3. Results

A total of 13,830 participants were included in the study, with a median age of 38 years (interquartile range (IQR): 26–51) for women and 39 years (IQR: 27–53) for men. Table 1 displays the characteristics of the participants. The prevalence of UI was 4.48%, ranging from 0.98% in the youngest age group (15–24 years) to 14.55% in the oldest age group (65+ years). UI was more prevalent in females (5.73%) than males (3.02%). In terms of residency status, rural residency was associated with a higher prevalence of UI (6.36%) compared to urban residency (3.66%). Alcohol consumption was categorized as “never”, “current user”, and “ex-user”. “Ex-user” was defined as a person who did not consume alcohol in the last 12 months. The highest UI prevalence was observed in the ex-user category (6.14%), while the lowest was seen in the current user category (1.93%) (Table 1).

While depression was observed in 4.21% of the population, mild, moderate, and high psychological discomfort were observed in 27.61%, 21.12%, and 30.33% of the population, respectively. Antidepressant use was reported by 2.10% of the population. Among those with depression, 16.84% reported UI, and the prevalence of UI was 11.38% among those who reported using depression medication. Similarly, anxiety prevalence was reported at 2.53%, and anxiolytic use at 1.33%. UI was reported by 20.29% of the population with anxiety and 11.96% of those using anxiety medication. Furthermore, a UI prevalence of 8.87% was observed in the subgroup with high psychological discomfort (Table 1).

**Figure 2:**
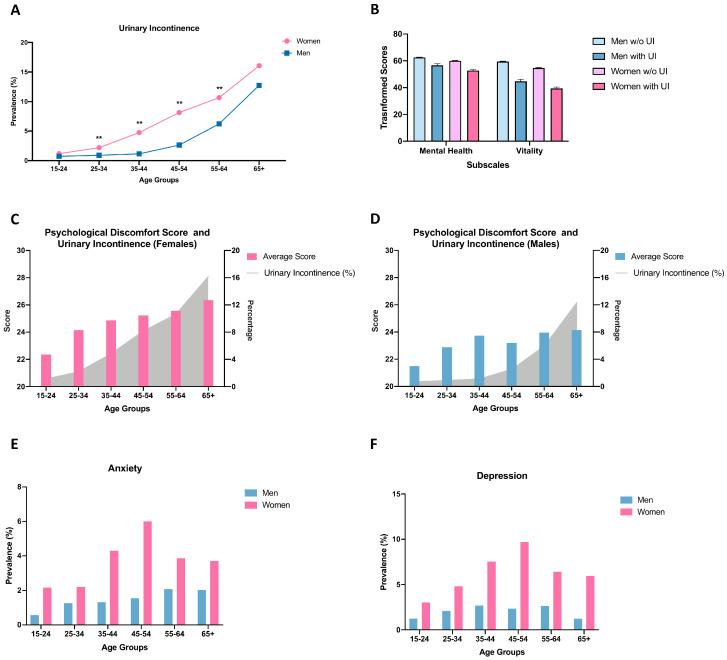
Comparison of UI, depression, and anxiety prevalence over ages between men and women. The prevalence of UI across ages in both sexes is depicted (**A**). Fischer’s exact test was used for the comparison of prevalence. ** *p* < 0.01. Mental health and vitality subscale scores were compared across genders and in relation to the presence or absence of UI (**B**). Average psychological discomfort scoring and UI prevalence overlapped for both sexes (**C**,**D**). Anxiety (**E**) and depression (**F**) prevalence were compared between genders.

The prevalence of UI increased in parallel with age. While the trends in both curves are similar, the prevalence is higher in females in all age groups. Additionally, the curve started to rise in women at a younger age (Figure 2A).

Mean scores for mental health and vitality subscales for both genders, with and without UI, were calculated. For both genders, individuals with UI had lower subscale scores compared to those without UI (Figure 2B). Additionally, the association of UI and gender with mental health and vitality was significant (*p* < 0.01, Supplementary Figure S1). The effect of the interaction was found to be insignificant (*p* = 0.20 for mental health, *p* = 0.79 for vitality).

The association between the mean psychological discomfort score and UI prevalence in different age groups is depicted in Figure 2C,D. This association is visibly stronger in females than in males.

In all age groups, depression and anxiety prevalence were higher in females than in males. Females aged 45–54 had the highest prevalence of both conditions. In contrast, males showed different patterns, with the highest depression prevalence in the 35–44 year age group and the highest anxiety prevalence in the 55–64 year age group (Figure 2E,F).

**Figure 3:**
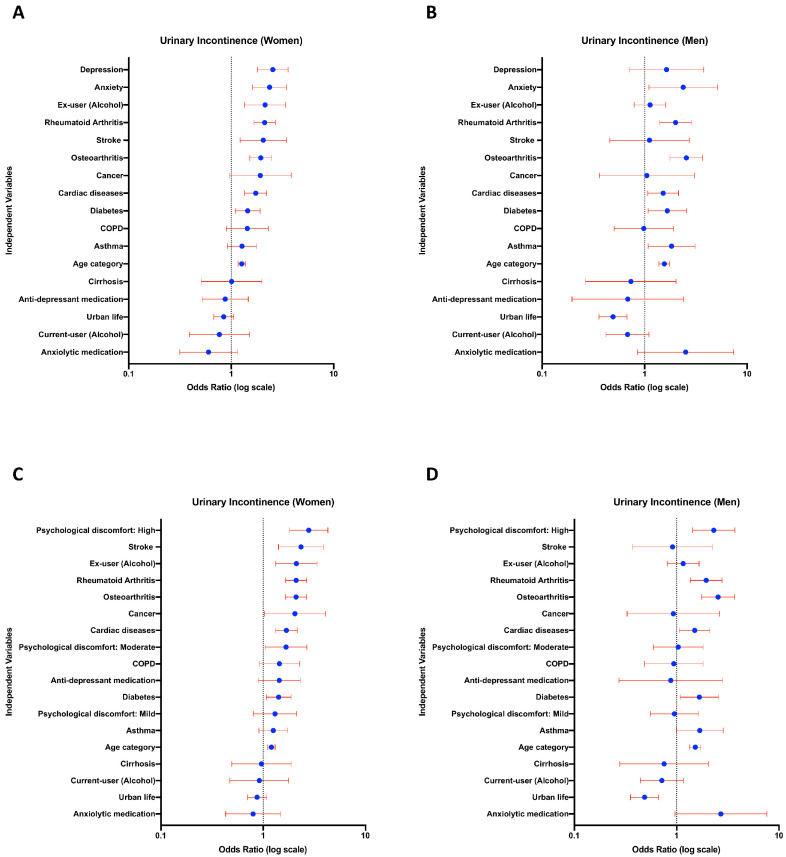
Anxiety and high psychological discomfort are significantly associated with urinary incontinence in both genders, while depression shows a significant relationship with urinary incontinence solely in women. Logistic regression analyses were conducted to examine the relationships between UI and depression, UI and anxiety (**A**,**B**), and UI and psychological discomfort (**C**,**D**). Analyses were carried out separately for each gender ((**A**,**C**) for women; (**B**,**D**) for men). Independent variables were sorted based on the odds ratios in women, and the same order was applied to men.

Gender-specific logistic regression models for anxiety and depression are shown in Figure 3. Based on these models, we conclude that anxiety was associated with UI, with ORs 2.36 (95% CI 1.61–3.46, *p* < 0.01) in females (Figure 3A) and 2.37 (95% CI 1.10–5.13, *p* = 0.02) in males (Figure 3B). Depression was also associated with UI in females with OR = 2.54 (95% CI 1.81–3.58, *p* < 0.01) (Figure 3A); however, this association, although with a similar trend, was not significant in males (OR = 1.63, 95% CI 0.71–3.76, *p*-value of 0.24) (Figure 3B). Furthermore, high psychological discomfort was found to be associated with a significantly higher likelihood of UI compared to no psychological discomfort with ORs of 2.78 in females (95% CI 1.80–4.29) (Figure 3C) and 2.30 in males (95% CI 1.43–3.71) (Figure 3D) with *p*-values less than 0.01. After adjusting for all known confounding factors, the use of medication for depression was not significantly associated with UI in either sex. Meanwhile, the relationship between the use of anxiety medication and UI exhibits contrasting trends in different genders.

## 4. Discussion

The present study demonstrates a strong association in both genders between high psychological discomfort and UI. Additionally, anxiety showed a significant association with UI in both sexes. Conversely, depression was significantly associated with UI in females, while it was not significant in males. Notably, the use of antidepressant and anxiolytic medications did not show a significant relationship with UI in either gender. However, there seems to be a trend in the adjusted models indicating that anxiety medication use has borderline significance for males. A similar trend was observed in females for depression medication use.

In a comprehensive cross-sectional study conducted by Felde et al., the prevalence of UI among females was estimated to range between 27.6 and 37.8% [20]. Conversely, the EPIC study estimated the global prevalence of UI in females to be 13.1% [34]. In the 2008 Turkish Health Studies survey, we estimated the prevalence of UI among females aged 15+ years to be 5.73%. Both national and international studies highlight significant variations in UI rates. The variability in prevalence might be attributed to cultural perceptions of UI, willingness to report it, studied population variances, and methodological differences such as the use of phone, in-person, or online surveys and different sampling procedures [4]. Although the joint report from the International Urogynecological Association/International Continence Society has provided a clear UI definition, some researchers still prefer to categorize prevalence based on the frequency of leakage, such as daily, weekly, monthly, or yearly occurrences [35]. Consequently, these factors make it difficult to consistently compare results across different population studies.

In the present study, the overall prevalence of UI was calculated as 4.48% in the study population and 3.02% in males. Our study’s broader age range and the fact that the survey size was determined to have sufficient representation in the 26 regions could explain why our results are more similar to global rates than those of population-based studies. Although there are fewer studies on the prevalence of UI in men than in women, nearly all community-based research indicates that the UI rates in men are lower than those in women, with a ratio of 1:2. A systematic review conducted by Buckley et al. outlined the prevalence of UI in males. The review reported a wide range of prevalence rates, varying from 1% to 39% across 21 studies. They found that UI was more prevalent in older men aged 65 years and above, with rates ranging from 11% to 34% [6]. However, Irwin et al. and Markland et al. conducted community-based studies on men in all age groups (starting from 18–20 years of age) and reported prevalence rates of 5.4% and 12.4%, respectively [34,36]. In accordance with these findings, the present community-based study involving men aged 15 years and older identified a lower prevalence of UI.

In line with the literature, women exhibit a higher prevalence of UI compared to men in all age groups, as observed in the present study (Figure 2A). The prevalence difference between men and women may be attributed to anatomical, hormonal, and functional factors. Women are exposed to physical stressors such as pregnancy and childbirth, which can weaken the pelvic floor muscles. On the other hand, men suffer from prostate-related issues, namely prostate enlargement resulting in lower urinary tract symptoms or complications following prostate surgery due to prostate cancer [6]. These differences also play a role in the different types of UI that are seen mostly in each sex. However, we could not specify the subtype of UI for either gender as this information was not collected in the survey. We are unclear about the weight of this missing information in our overall analysis. For example, in females, stress urinary incontinence (SUI) is the most prevalent type and may potentially influence their psychological status [37]. Conversely, psychological factors do not contribute to stress urinary incontinence since it primarily stems from a weakened pelvic floor. Therefore, the connection between stress urinary incontinence and psychological issues is believed to be unidirectional. In contrast, there is a belief that patients with urge urinary incontinence (UUI) might experience more significant psychological challenges than those with stress urinary incontinence, given that urge urinary incontinence has a bidirectional relationship with mental health [37]. While various studies have highlighted the differences in how different UI types interact with psychological issues, none have explored the reasons for these disparities [38,39]. This gap suggests a potential direction for future research to explore the underlying mechanism for relationships of psychological diseases with different UI types.

Differences between genders were also observed in the logistic regression model outcome controlling for other comorbidities. As illustrated in Figure 3, depression and anxiety are the top two independent variables with the highest odds ratios related to UI in females. Moreover, a significant association between UI and factors such as depression, stroke, and past alcohol use was observed exclusively in women. On the other hand, asthma and living in rural areas were only significant in men. Age, anxiety, cardiac diseases, diabetes, rheumatoid arthritis, and osteoarthritis are all significantly associated with UI in both sexes with different magnitudes.

The mechanism underlying the gender disparities in the relationship between mental health problems and UI has not yet been clearly identified. However, one study pointed out that serotonin synthesis is 52% higher in males than in females [27]. This study hypothesized that decreased serotonin activity might explain why bladder overactivity and major unipolar depression are more common in females. Furthermore, it was suggested that a shared neurochemical abnormality could underpin both incontinence and depression. Such insights could be pivotal for screening, prevention, or crafting new pharmacological treatments.

Several other studies have confirmed the relationship between UI and psychological discomfort. It goes beyond saying that psychological discomfort has many different dimensions. According to The Global Burden of Diseases, Injuries, and Risk Factors Study (GBD), depressive and anxiety disorders are the two most impairing mental health conditions and are ranked among the top 25 leading causes of global burden [40,41]. This burden was substantial throughout all age groups, affecting both sexes and across many locations. According to cross-sectional comparisons, the prevalence estimates of depression vary substantially among countries. The lifetime prevalence ranges from 1.0% to 19%, and the 12-month prevalence ranges in different studies from 0.3% to 10% [42]. Over the course of a lifespan, depression, and anxiety are nearly twice as common in women than in men. In both genders, the highest prevalence occurs during middle age and then tends to decline with aging [42,43,44,45]. In the present study, we found that the prevalence of depression was 6.03% in Turkish women and 2.06% in Turkish men. In accordance with previous findings, a rising trend was noted in depression prevalence amongst women as they approached middle age. The highest peak occurs around the fourth and fifth decades. Interestingly, this peak appeared during the fifth and sixth decades in the 2008 global estimates of depression prevalence [41], which might be explained by socioeconomic and cultural differences. Similar to previous reports, the distribution of lifetime depression and anxiety in men was fairly evenly spread across the age groups [46]. But anxiety showed a slight increase over 55 years of age. The disparities in depression and anxiety prevalence between men and women are known as the gender gap in depression and anxiety and are attributed to hormonal, genetic, and environmental factors, including gender inequity [44,45].

The findings regarding the connection between UI, anxiety, and depression in females correspond well with previous cross-sectional studies. A population-based study among 21,803 women showed that severe anxiety and depression, as measured by the Hospital Anxiety and Depression Scale (HADS), were significantly associated with UI [13]. Similarly, a meta-analysis by Cheng et al. found that populations with UI had significantly higher depression and anxiety levels [29]. In the present study, we investigated the relationship between the severity of psychological discomfort and UI in greater detail using the SF-36 psychological discomfort scale. A strong association in both females and in males was found.

Markland et al. conducted the first national survey study that identified the factors associated with moderate to severe UI in men and revealed a substantial correlation between UI and major depression [36]. Our logistic regression analysis largely supports their findings. However, the present study also examined anxiety separately from depression and included a wider variety of comorbidities. In contrast to Markland et al., we could not observe a significant relationship between UI and depression in men. But the presence of depression did increase the odds ratio of having UI. In a cross-sectional online survey, Coyne et al. found that men over 40 with UI experienced an increased mental health burden [23]. Confirming their results, our study validated this association across a wider age range using a more extensive, government-led survey.

The two SF-36 subscales, mental health, and vitality, are found to be highly correlated with psychological well-being [32]. Especially, low levels of mental health scores are suggested to be a predictor of depression. According to some studies, UI lowers all 8 subscales of the SF-36, including mental health and vitality. In contrast, other studies found that UI does not affect mental health and vitality significantly [47,48]. In this study, we found that patients with UI had lower mental health and vitality scores than those without UI, with women generally scoring lower than men (Figure 2B). However, this decline in the mental health and vitality subscale scores is not exclusive to UI. Similar trends were also observed in patients with cardiac diseases, diabetes, and stroke (Supplementary Figure S1). Thus, the decrease in these scores could be influenced by the presence of any significant health condition.

The effect of antidepressants and anxiolytics on UI remains controversial [49,50,51]. The factors such as the type of UI, gender, and the specific mechanisms through which certain antidepressants and anxiolytics interact with the bladder may play a role in the development of involuntary urine leakage. In this study, we did not observe any association between UI and psychotropic medication use, which is consistent with the findings of Felde et al. (Figure 3A,B) [13]. Additionally, medication use in patients with depression or anxiety did not significantly alter the prevalence of UI compared to non-users in either sex (Supplementary Table S1). However, the low number of drug use in patients with UI and psychological distress may have impacted the statistical significance.

The present study has several limitations. First of all, our data suffer from the so-called chicken-and-egg issue in the relationship between depression, anxiety, and UI. Since this is a cross-sectional study, it does not allow us to provide causality between conditions. Furthermore, the types of UI for both genders and parity in women were not included in the survey, which could have provided more insight into the relationship between UI and psychological discomfort in females. Additionally, the presence of both mental conditions and UI were stated via patient-reported outcomes (PROs) and irrespective of a doctor’s confirmation or formal diagnosis.

The strengths of our study include being the largest and first population-based study in Turkiye to investigate the relationship between UI, depression, and anxiety in both genders. The study included all age groups and was organized by a governmental institute (TurkStat) in accordance with Eurostat, which allowed for a high response rate and concordance with the population demographics of 2008. Previous studies conducted in the Turkish population have primarily focused on females and have been conducted regionally or within hospital settings, which may have affected the accuracy and completeness of their findings.

In one of the most comprehensive Turkish studies, the objective was to demonstrate the prevalence and risk factors of UI using a standardized survey through house visits. However, the study was restricted to a single city and only included women [9]. Another Turkish study aimed to determine UI prevalence in women by selecting participants from primary care physicians’ patient lists [52]. As a result, only those who visited primary care centers were included in the survey, potentially narrowing the population sample and overestimating the prevalence. Additionally, a Turkish study explored the relationship between UI and depression, but it focused solely on elderly patients over 65 years using limited hospital-based data [53]. These limitations in previous Turkish studies may have led to a higher calculated prevalence when compared to other national and international studies.

In contrast, our study had a larger sample size, a greater number of independent variables, and a more comprehensive approach, which provides a more representative and reliable depiction of the Turkish population. This study contributes to the importance of the relationship between psychological distress and UI in men across all age groups, an issue that a limited number of studies pointed out before. The survey included numerous questions, which may have helped to decrease the stigma problem often associated with discussing involuntary urine leakage. Lastly, a vast list of confounders and demographics was included in the logistic regression model, which increases the reliability and validity of our findings.

## 5. Conclusions

The primary outcome showed no overall significant gender differences between the association of depression and anxiety with urinary incontinence (UI). While depression has a significant relationship with UI in females, its association with UI in males is not as pronounced. Similarly, no gender differences were found in the correlation between psychological discomfort and UI prevalence. Conversely, high psychological discomfort has a strong association with UI in both sexes. The use of antidepressants was not found to be significantly associated with UI in either sex. However, the relationship between the use of anxiolytics and UI displays divergent trends among the genders. Lastly, our findings for the Turkish population are in line with the outcomes from similar international studies.

These findings emphasize the importance of a holistic approach to managing UI in individuals with mental health conditions. Healthcare providers should be aware of the strong link between UI, depression, and anxiety and consider addressing these psychological factors when developing treatment plans for patients with UI. It is also crucial not to overlook the need for a comprehensive approach for males while considering the gender disparity and relatively faster incline of UI among males of older ages. This calls for the creation of multidisciplinary teams, including urologists, gynecologists, physiotherapists, radiologists, and psychologists. Further research is needed to better understand the underlying mechanisms that contribute to the relationship between psychological distress and UI, as well as to explore potential interventions that could reduce the burden of UI in individuals with mental health conditions.

## Figures and Tables

**Table 1:** Characteristics of the study population (N = 13,830). All participants and participants reporting UI are presented separately. Individuals with any of the following cardiovascular conditions: myocardial infarction, hypertension, chronic heart failure, or coronary artery disease, were categorized as having cardiac disease.

Participants	All (N = 13,830)		UI (N = 620)	
	N	%	N	% of Total
**Gender**				
● Male	6355	45.95	192	3.02
● Female	7475	54.05	428	5.73
**Age Distribution (years)**				
● 15–24	2757	19.93	27	0.98
● 25–34	3158	22.83	51	1.61
● 35–44	2741	19.82	86	3.14
● 45–54	2271	16.42	124	5.46
● 55–64	1501	10.85	128	8.53
● 65+	1402	10.14	204	14.55
**Residency Status**				
● Rural	4217	30.49	268	6.36
● Urban	9613	69.51	352	3.66
**Asthma**	692	5.00	86	12.43
**COPD**	299	2.16	48	16.05
**Myocardial Infarct**	260	1.88	55	21.15
**Coronary Heart Disease**	889	6.43	131	14.74
**Chronic Heart Failure**	334	2.42	53	15.87
**Hypertension**	2196	15.88	275	12.52
**Cardiac Disease (any)**	2753	19.906	337	12.24
**Cerebral Stroke**	165	1.19	33	20.00
**Osteoarthritis**	1782	12.89	292	16.39
**Rheumatoid Arthritis**	2504	18.11	340	13.58
**Diabetes Mellitus**	897	6.49	133	14.83
**Cancer (any)**	120	0.87	19	15.83
**Alcohol Consumption**				
● Never	10,857	78.50	507	4.67
● Ex-user	1319	9.54	81	6.14
● Current user	1654	11.96	32	1.93
**Liver Cirrhosis**	150	1.08	18	12.00
**Anxiety**	350	2.53	71	20.29
**Depression**	582	4.21	98	16.84
**Psychological Discomfort Score**				
● Normal	2897	20.95	49	1.69
● Mild	3818	27.61	91	2.38
● Moderate	2921	21.12	108	3.70
● High	4194	30.33	372	8.87
**Anxiety Medication**	184	1.33	22	11.96
**Depression Medication**	290	2.10	33	11.38
**Urinary Incontinence**	620	4.48		

## Data Availability

The data used in this study were taken from the Turkish Statistical Institute (TurkStat), but the accessibility of these data is limited due to certain constraints. The data were licensed specifically for this study and are not publicly available. However, authors can provide the data upon a reasonable request and with included permission from TurkStat, and the Regional Ethical Committee.

## Supplementary Materials

The following supporting information can be downloaded at: https://www.mdpi.com/article/10.3390/jcm12175535/s1, Figure S1: Mental health and vitality subscale scores were compared across genders and in relation to the presence or absence of diseases. Two-way ANOVA test were used for statistical analysis. MH: Mental Health, VT: Vitality, UI: Urinary incontinence; Table S1: The effect of antidepressant or anxiolytic medication use on the relationship between urinary incontinence and depression/anxiety. A logistic regression model was utilized to explore how the usage of antidepressants or anxiolytic medications influences the association between urinary incontinence and conditions of depression or anxiety, respectively. The medication use did not show any significant relation with urinary incontinence among people with depression or anxiety.
