# Supplementary material for: When Bladder and Brain Collide: Is There a Gender Difference in the Relationship between Urinary Incontinence, Chronic Depression, and Anxiety?
Source: J Clin Med. 2023 Aug 25;12(17):5535. doi: 10.3390/jcm12175535 (PMC10488595; doi:10.3390/jcm12175535)
Supplement: Supplementary file 1 [file jcm-12-05535-s001.zip › jcm-2534567-supplementary.pdf]

Supplementary Table 1: The effect of antidepressant or anxiolytic medication use on the relationship of urinary incontinence and depression/anxiety. A logistic regression model was utilized to explore how the usage of antidepressants or anxiolytic medications influences the association between urinary incontinence and conditions of depression or anxiety, respectively. The medication use did not show any significant relation with urinary incontinence among people with depression or anxiety.

		N	%	Adjusted OR	P value
WOMEN	No depression	341	0.05	0.31 (0.22 – 0.43)	0.00
	Depression without use of anti-depressants	64	0.21	1.00 (ref.)	
	Depression with use of anti-depressants	23	0.16	0.69 (0.39 – 1.23)	0.21
	No anxiety	371	0.05	0.30 (0.21 – 0.43)	0.00
	Anxiety without use of anxiolytics	49	0.22	1.00 (ref.)	
	Anxiety with use of anxiolytics	8	0.20	0.71 (0.28 – 1.80)	0.46
MEN	No depression	181	0.03	0.35 (0.16 – 0.75)	0.01
	Depression without use of anti-depressants	10	0.11	1.00 (ref.)	
	Depression with use of anti-depressants	1	0.03	0.21 (0.02 – 1.90)	0.16
	No anxiety	178	0.03	0.34 (0.16 – 0.71)	0.00
	Anxiety without use of anxiolytics	12	0.15	1.00 (ref.)	
	Anxiety with use of anxiolytics	2	0.25	1.15 (0.14 – 9.40)	0.90

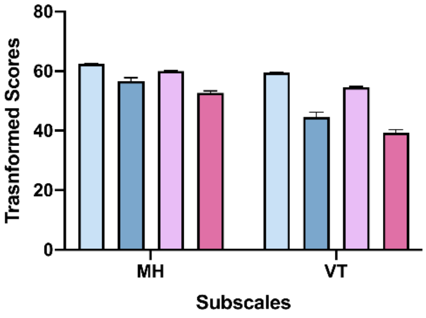

	Effect of UI	Effect of Gender	Effect of Interaction
MH	<0.01	<0.01	0.2017
VT	<0.01	<0.01	0.7910

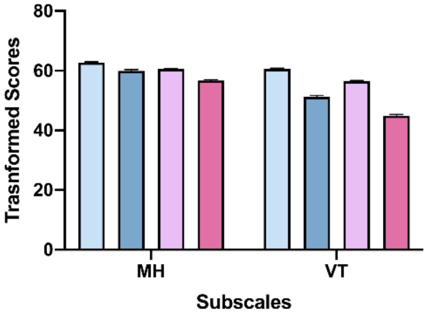

	Effect of Cardiac Dis	Effect of Gender	Effect of Interaction
MH	<0.01	<0.01	0.0936
VT	<0.01	<0.01	0.0080

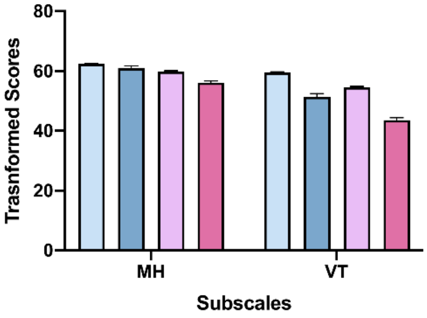

	Effect of Diabetes	Effect of Gender	Effect of Interaction
MH	<0.01	<0.01	0.0181
VT	<0.01	<0.01	0.0251

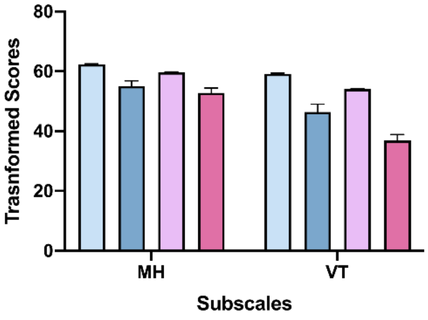

	Effect of Stroke	Effect of Gender	Effect of Interaction
MH	<0.01	0.0243	0.8478
VT	<0.01	<0.01	0.1581

Figure S1. Mental health and vitality subscale scores were compared across genders and in relation to the presence or absence of diseases. Two-way ANOVA test were used for statistical analysis. MH: Mental Health, VT: Vitality, UI: Urinary incontinence
